# Health innovations to achieve universal health coverage in the Western Pacific region

**DOI:** 10.1016/j.lanwpc.2023.101000

**Published:** 2023-12-28

**Authors:** The Lancet Regional Health – Western Pacific

WHO defines universal health coverage (UHC) as “all people obtain the health services they need without suffering financial hardship”. According to the *Tracking Universal Health Coverage: 2023 Global Monitoring Report* released by WHO and the World Bank on Sept 23, 2023, the world is not on the path towards achieving UHC by 2030 as 4.5 billion people were not covered by essential health services, and an increasing number of people are facing out-of-pocket spending on health. Despite the progress that has been made in the Western Pacific region, improvement in health service coverage has stagnated since 2015. The prevalence of catastrophic spending has doubled from one in ten to one in five people between 2000 and 2017.

Countries in the Western Pacific region are at different stages of UHC in terms of service coverage and financial management, but some similar challenges exist in implementing UHC. These challenges include rapid population ageing and a surge of non-communicable diseases; fragmented health service delivery as people bypass primary care and crowd in hospitals for curative care; and the persistence of health inequities in rural areas and for socioeconomically disadvantaged groups. Overcoming these challenges requires huge financial investments and will take many years, which may cause these countries to ultimately fall short of the Sustainable Development Goals targets by 2030. How can the Western Pacific region meet these challenges? In a workshop on developing sustainable UHC, organised by Duke Kunshan University in Oct, 2023, researchers from China and other countries gathered to discuss challenges and innovations to achieve UHC in the Asia–Pacific region and the world.

It is vital to have robust health information systems that provide timely and accurate data to inform policy development, allocate resources, and monitor the impact of interventions to achieve UHC. Professor Peng Jia and colleagues from Wuhan University created maps of the spatial accessibility of primary health-care centres in China by collecting data on administrative boundaries, residential communities, primary health-care centre locations, and road networks. This study reveals the spatial inequality of primary health-care centres in China and provides evidence for precise health-care planning. According to the *Tracking Universal Health Coverage: 2023 Global Monitoring Report*, the availability of primary data to monitor the progress of UHC in the Western Pacific region is 70%, while the data from the African and South-East Asia regions are 80% and 84%, respectively. Health data from many Pacific Island countries covers less than half of UHC indicators. Besides, disaggregated data to monitor unmet health-care needs and other health inequalities, non-communicable diseases, mental health, and injuries are limited. The Western Pacific region is experiencing the expansion of the private health sector; for instance, the number of private hospitals surpassed public hospitals in China in 2015. Health services in the private health sector generate enormous amounts of data but these are not routinely collected. The value of data is hampered by disconnected systems, a lack of standardisation, and limited analytical capacity, which makes it difficult to integrate data from multiple sources to meet individualised health needs. Issues on data quality and availability require further investment in health information systems. Dr Kidong Park from the WHO Western Pacific regional office proposed implementing a digital health strategy in the region to achieve UHC. Although the digitalisation of health information systems has its strengths in improving the efficiency and quality of health systems, there is still a pressing need to improve infrastructure, human resources, and management of digitalised health information systems, especially in low-resource settings.

The quality and availability of health workers are core elements of a well performing health system. The Western Pacific region is extremely short of qualified health workers to address emerging health needs related to population ageing and non-communicable diseases. According to the *Regional Framework to Shape a Health Workforce for the Future of the Western Pacific* released by the WHO Western Pacific regional office on Oct 20, 2023, health workers with knowledge and skills in disease prevention and health promotion, and who provide integrated and long-term care, are especially needed. In addition, health workforce density in eight Western Pacific countries is below the global median of 49 per 10,000 people. One possible solution is to optimise the practice scope of the existing health-care workers. For instance, Michele Rumsey and colleagues call for leadership from nurses and midwives in policy making in Pacific Island countries to achieve UHC. But in the long run, reforms in medical education are needed, especially for low-income and middle-income countries where the traditional medical education models have been criticised for outdated curricula, not meeting population health needs, and having limited continuing professional development. Professor Tien Yin Wong from Tsinghua University described the potential for artificial intelligence (AI) technology integrated into medical education and clinical practice to provide life-long learning opportunities for physician and non-physician health-care workers.

Digital health and AI technologies have been applied in almost every part of health practice. Professor Dame Margaret Whitehead from the University of Liverpool warned that AI might exacerbate existing health inequities regarding the accessibility and the performance of effective digital health services for different groups. We are increasingly becoming aware of the inequity of accessing new technologies, but the biased performance of new technologies and devices is often invisible and difficult to detect. For example, pulse oximeters provide less accurate readings in people with darker skin as there were fewer tests in minoritised ethnic populations. The performance of AI algorithms and other digital devices might not be effective in women and people of lower socioeconomic status who are under-represented in data sources. To maximise the potential of digital health and AI technologies in health-care systems, concerted actions are needed to increase transparency and regulation of new technology to have more equitable and bias-free digital and AI applications.

The theme of this year's World UHC Day on Dec 12, 2023, is Health for All: Time for Action. The progress towards achieving UHC in the Western Pacific region is uneven, requiring context-specific policies and solutions for every country. Digital solutions show potential to improve the quality, accessibility, and affordability of health services; we need to take the power of digital innovations and other forms of innovation to achieve the goal of having equitable access to quality health services without financial hardship in the Western Pacific region.

